# Comparative Effectiveness of Biologic Agents Among Black and White Medicare Patients in the US With Metastatic Colorectal Cancer

**DOI:** 10.1001/jamanetworkopen.2021.36378

**Published:** 2021-12-15

**Authors:** Sanjay Goel, Abdissa Negassa, Ana Acuna-Villaorduna

**Affiliations:** 1 Department of Medical Oncology, Montefiore Medical Center, Albert Einstein College of Medicine, Bronx, New York; 2 Department of Epidemiology and Population Health, Albert Einstein College of Medicine, Bronx, New York

## Abstract

**Importance:**

Randomized clinical trials have defined the survival benefit provided by the addition of biologic drugs to chemotherapy in patients with metastatic colorectal cancer (mCRC). However, Black patients may be underrepresented in trial populations, and outcomes in this group remain poorly defined.

**Objective:**

To determine whether the real-world benefit of biologic drugs in Black patients is consistent with the real-world benefit of biologic drugs in White patients using a comparative effectiveness research approach.

**Design, Setting, and Participants:**

Population-based retrospective comparative effectiveness analysis of a cohort of patients aged 65 years or older with mCRC diagnosed between 2004 and 2011 who had received at least 1 dose of chemotherapy and had complete Medicare claims data using the Surveillance, Epidemiology, and End Result (SEER)–Medicare linked database. Data were analyzed from August 1, 2020, to March 31, 2021.

**Interventions:**

Patient data were classified according to whether patients received chemotherapy (oxaliplatin, irinotecan, and 5-fluorouracil or capecitabine) or biochemotherapy (bevacizumab, cetuximab, panitumumab, ramucirumab, or aflibercept, started within 3 months of chemotherapy).

**Main Outcomes and Measures:**

Overall survival (OS) defined as the time from starting chemotherapy to death or last follow-up. A weighted Cox regression model was used to assess differences in survival.

**Results:**

A total of 5617 patients with mCRC were identified in the SEER-Medicare linked database, and 4542 patients were included in the main analysis. Of the 5617 patients, 3969 (70.7%) received biologic agents at any point between 2004 and 2011; biologic agent therapy was started within 3 months of chemotherapy in 2894 patients (72.9%). Among 4542 patients with data on race and ethnicity, the median age was 72 years (IQR, 68-78 years), 2365 (52.0%) were female, 3445 (75.8%) had colon as the primary site, 552 (12.2%) were Black patients, and 3990 (87.8%) were White patients. There was no difference in the receipt of 1 (76.7% and 74.8%) vs 2 or more (23.3% and 25.2%: *P* = .92) lines of therapy, and in the receipt of biologic agents (63.6% vs 64.3% *P* = .33), among White patients and Black patients, respectively. Biochemotherapy was associated with a significant survival benefit compared with chemotherapy alone in the overall population (biochemotherapy median OS, 17.9 [95% CI, 17.3-18.7] months vs chemotherapy median OS, 8.3 [95% CI, 9.1-9.9] months; *P* < .001). The survival benefit was similar among White patients (17.8 vs 9 months; average hazard ratio, 0.59; 95% CI, 0.55-0.64; *P* < .001) and Black patients (18.6 vs 9.9; average hazard ratio, 0.58; 95% CI, 0.47-0.71; *P* < .001).

**Conclusions and Relevance:**

In this comparative effectiveness analysis of a cohort of Medicare recipients with mCRC, biochemotherapy was associated with an improvement in OS with a similar rate of reduction in mortality among Black and White patients. Clinicians may offer biochemotherapy therapy to all patients to maximize clinical benefit, factoring in clinical variables, but not their race.

## Key Points

**Question:**

Is the addition of biologic drugs to chemotherapy associated with improved survival in Black patients with metastatic colorectal cancer?

**Findings:**

In this comparative effectiveness analysis of a cohort of 2894 patients who received biologic agents within 3 months of chemotherapy (of the 4542 patients diagnosed with metastatic colorectal cancer between 2004 and 2011), Black patients experienced a survival benefit from the addition of biologic agents to cytotoxic chemotherapy similar to that of White patients.

**Meaning:**

The results of this study suggest that biologic agents have the same utility for patients who identify as Black as they do for those who identify as White.

## Teaser

This comparative effectiveness analysis assesses whether the overall survival benefit of biologic drugs in White patients is similar to that for Black patients in a real-world setting using the Surveillance, Epidemiology, and End Result (SEER)–Medicare linked database.

## Introduction

Colorectal cancer (CRC) is expected to be diagnosed in 149 500 US adults and result in the death of 52 980 US adults as of 2021.^1^ At presentation, 21% have metastatic CRC (mCRC),^1,2^ with an expected 5-year overall survival (OS) of 14% to 15%.^3,4^ The standard therapy includes the cytotoxic drugs 5-fluorouracil, capecitabine, oxaliplatin, and irinotecan.^2^ Since 2004, biologic drugs, targeting the vascular endothelial growth factor (bevacizumab,^5^ ramucirumab,^6^ aflibercept,^7^ and regorafenib^8^) and the epidermal growth factor receptor (cetuximab^9,10^ and panitumumab^11^) have been approved by the US Food and Drug Administration. In randomized clinical trials (RCTs), each has shown a statistically significant and clinically meaningful improvement in OS when added to chemotherapy.^5,6,7,8,9,10,11^ However, individuals who identify as belonging to racial and ethnic minority groups are projected to comprise more than 50% of the US population by 2045.^12^ This population is underrepresented in RCTs, with, as of 2019, more than 90% of patients enrolled in cancer RCTs being White individuals.^13,14^

The use of bevacizumab exemplifies the disconnect between RCT data and the real-world experience. The greatest benefit of bevacizumab, shown in an RCT,^5^ was seen when added to the irinotecan, fluorouracil, and leucovorin regimen for metastatic colorectal cancer (OS benefit of 4.7 months). When added to folinic acid/5-FU/oxaliplatin (FOLFOX) in the first-line therapy,^15^ the benefit was more modest (OS benefit of 1.4 months) as it was in second-line^16^ therapy (OS benefit of 2.1 months). Similarly, a population-based study, using the Surveillance, Epidemiology, and End Results (SEER)–Medicare linked database, reported that the addition of bevacizumab was associated with a more modest improvement in OS, which was restricted to its use with irinotecan.^17^ Despite this finding, FOLFOX continues to be the preferred front-line regimen, prescribed by 64% of US oncologists.^18^

Previously, a large RCT^19^ evidenced racial and ethnic disparities in the OS rates with bevacizumab added to FOLFOX chemotherapy (OS for Black patients, 10.2 months vs OS for NHW patients, 11.8 months; *P* = .003). Similarly, a single-center comparative effectiveness research study reported a survival benefit with biochemotherapy compared with chemotherapy (25.6 vs 15.2 months), which was statistically significant among White patients; findings for Black patients and Hispanic patients were not statistically significant.^20^ As we acknowledge the limitation of a single-center retrospective study, we sought to confirm this observation using a large national database.^21,22,23^

This study goal was to assess whether the OS benefit of biologic drugs in Black patients is consistent with the OS benefit of biologic drugs in White patients in a real-world setting using the SEER-Medicare linked database.

## Methods

### Patient Selection and Characteristics

This population-based retrospective cohort study included patients diagnosed with mCRC between 2004 and 2011, identified using the SEER-Medicare linked database. Data were analyzed from August 1, 2020, to March 31, 2021. For inclusion, beneficiaries were required to be diagnosed with mCRC, with at least 13 continuous months of coverage in Part A and B or Part D, with no enrollment in Medicare managed care. Only participants who had received at least 1 dose of chemotherapy for treatment of mCRC were included. With the goal of assessing findings in Black and White patients, patients with race and ethnicity other than Black or White race were excluded (ie, American Indian/Alaska Native, Asian/Pacific Islander, and Hispanic). This study was approved with a waiver of informed consent owing to study type by the Ethics Committee of Albert Einstein College of Medicine. This study followed the relevant portions of the International Society for Pharmacoeconomics and Outcomes Research (ISPOR) reporting guideline as well as the Strengthening the Reporting of Observational Studies in Epidemiology (STROBE) reporting guideline as recommended by ISPOR.

### Data Extraction

Data were extracted from the SEER-Medicare linked database. Medicare insures more than 96% of the US population aged 65 years and older. Of this population, approximately 95% of cases are matched to the SEER cancer registry using Medicare administrative files from the inpatient and outpatient settings. Overall, the SEER-Medicare linked database represents 28% of the US population from 20 geographic regions. Patient data are generated as a collaboration between the SEER registries, Centers for Medicare & Medicaid Services, and the National Cancer Institute. Data include demographic characteristics (age, race and ethnicity [self-reported in SEER], and median income [derived from census track data provided by SEER]), clinical features (date of diagnosis, cancer site, stage of disease, site of origin of tumor, histology, degree of differentiation), treatment (use of chemotherapy and biologic agents), and outcomes (date of death or last follow-up).

Race and ethnicity information was obtained directly from SEER-Medicare using the Patient Entitlement and Diagnosis Summary File which has a reportedly 94% match with Medicare enrollment records. To calculate the Charlson Comorbidity Index score, claims from 13 months before and 1 month after mCRC diagnosis were reviewed to gather information about preexisting conditions, and the calculated Charlson Comorbidity Index score was classified as 0, 1, or 2 or greater.^24,25^ Census-tract median income served as a proxy for socioeconomic status. Chemotherapy and biologic treatment information was obtained using J codes for 4 chemotherapy drugs (5-FU, capecitabine, oxaliplatin, and irinotecan), and 5 biologic agents (bevacizumab, cetuximab, panitumumab, ramucirumab, and aflibercept).

### Definitions

Patients were classified as being in the chemotherapy group if they had received any chemotherapy but had not received any biologic agent and in the biochemotherapy group if they had received any biologic agent within 3 months of the first dose of chemotherapy. The primary end point was OS, defined as the interval between first dose of chemotherapy and date of death or last follow-up, whichever occurred first. Patients who were alive at the time of access to the SEER database were censored at the time the patient was last alive per SEER data through December 31, 2013.

### Statistical Analysis

Baseline characteristics were summarized using descriptive statistics; median with IQR for continuous variables and frequency with percentage values for discrete variables. For comparison of groups with respect to baseline characteristics, *t* test or nonparametric equivalent for continuous variables and χ^2^ tests for discrete variables were used. OS was examined using a nonparametric approach^26^ and a weighted Cox regression model, with biochemotherapy treated as a time-dependent covariate. Model assumptions of proportional hazards were assessed with plots of Schoenfeld residuals vs time as well as a formal test for proportionality.^27^ An inverse probability of treatment weighting approach was undertaken to further account for potential treatment selection bias.^28^

#### Imputation Model

Multiple imputations using the algorithm of full conditional specification were used to incorporate the extra variability induced from the imputation.^29^ Essentially, rather than imputing a single value for participants with missing covariates, we generated 50 values. For each realization, the corresponding set of complete data were analyzed in a standard manner, and the results were pooled using a set of rules proposed by Rubin.^30^ The imputation method regressed each covariate with a missing value using an a priori specified set of covariates and the outcome of interest. Next, random draws were taken from the posterior predictive conditional distribution. An assumption in multiple imputations is that of missing at random,^30^ which indicates that the probability of missingness could depend on data that were observed but not on missing data values. In the SEER-Medicare database, missing at random was a reasonable assumption.

Three covariates had missing values, with a varying level of missingness: marital status (2.9%), degree of differentiation (16.4%), and median income (2.0%). There was no systematic pattern in missing data.

#### End Point Model

We used a weighted Cox regression model^31^ with stabilized inverse probability of treatment weighting to estimate the effectiveness of biologic agents in OS using robust standard errors. The stabilized weights were computed at each event-time, as described earlier,^28^ considering month as the time-unit, up to the time of first treatment (ie, treatment as a time-dependent covariate). Based on an a priori specification of potential confounding and association with treatment assignment status, the covariates age, sex, race and ethnicity, census tract median income, degree of differentiation, colon/rectal primary, sidedness of primary cancer, Charlson Comorbidity Index score, marital status, lines of chemotherapy (treated as a time-dependent covariate), and year of diagnosis were included in the derivation of stabilized weights. Stabilized inverse probability of treatment (biochemotherapy) weighting was used to estimate the effectiveness of the biologic agents by appropriately adjusting for confounding and treatment selection bias. We also checked the stabilized weights and few were extreme (ie, <0.1 or >10). Those few instances with extreme inverse probability of treatment weightings were excluded from subsequent analysis.

In all analyses, biochemotherapy was treated as a time-dependent covariate to avoid the possibility of immortal time bias.^32^ Therefore, within the first 3 months, the time before initiating biologic agents for each individual was appropriately attributed to chemotherapy. The a priori specified interaction term (the interaction between biochemotherapy and race) was assessed, followed by the subgroup analysis. The results are presented as the point estimate of average effectiveness of biochemotherapy or average hazard ratios (HRs) and associated 95% CIs.

As an exploratory analysis, a dynamic modeling approach^33^ to assess the possible change in the effect of biochemotherapy over time and a piecewise weighted Cox regression model were used to further describe the time-varying effects of biochemotherapy.

A sensitivity analysis was conducted to assess an alternate handling of initiation of biologic agents late in the follow-up (ie, after 3 months) by censoring at the time of biologic agent initiation and using inverse probability of censoring weighting to account for the possibility of selection bias owing to the artificial censoring. In addition to the nominal computed *P* value, we also computed multiple comparison-adjusted *P* values using the Benjamini and Hochberg method.^34^ The primary results are based on the adjusted *P* values and considered statistically significant at 2-sided *P* < .05. All analyses were performed using the SAS software version 9.4 (SAS Institute Inc) or R version 3.4.1 (R Foundation for Statistical Computing).

## Results

### Patient Characteristics

Among 30 849 patients diagnosed with CRC, 5617 (18.2%) had mCRC and 3969 (70.7%) received biologic agents between 2004 and 2011. Of this population, therapy with biologic agents was started within 3 months of chemotherapy in 2894 patients (72.9%) (eTable 1 in the Supplement). Table 1 presents the baseline characteristics of 4542 patients by race and ethnicity. Participants had a median age of 72 years (IQR, 68-78 years), 2365 (52.0%) were female, 3445 (75.8%) had colon as the primary site 552 (12.2%) were Black patients, and 3990 (87.8%) were White patients. There was no difference in the receipt of 1 (76.7% vs 74.8%) vs 2 or more lines of therapy (23.3% vs 25.2%; *P* = .92), and in the receipt of chemotherapy or biologic agents (63.6% vs 64.3%; *P* = .33) between Black and White patients (Table 1; eTables 2 and 3 in the Supplement).

**Table 1.** Baseline Characteristics by Race in 4542 Patients^a^

Characteristic	No. (%)		*P* value
	White race	Black race	
Total No.	3990 (87.8)	552 (12.2)	
Age, median (IQR), y	73 (68-78)	70 (66-75)	<.001
Sex			
Female	2111 (52.9)	254 (46)	.002
Male	1879 (47.1)	298 (53)	
Census tract median income^b^			
First quartile	832 (20.8)	285 (51.6)	<.001
Second quartile	1023 (25.6)	131 (23.7)	
Third quartile	972 (24.4)	67 (12.3)	
Top quartile	1085 (27.2)	56 (10.1)	
Unknown	78 (2)	13 (2.3)	
Marital status			
Single	326 (8.2)	101 (18.3)	<.001
Married	2344 (58.7)	208 (37.8)	
Separated	12 (0.3)	20 (3.6)	
Divorced	367 (9.2)	67 (12.1)	
Widowed	820 (20.6)	141 (25.5)	
Unknown	121 (3)	15 (2.7)	
Grade of differentiation			
Well/moderate	2268 (56.8)	343 (62.1)	<.001
Poor	1051 (26.4)	105 (19)	
Unknown/NA	671 (16.8)	104 (18.9)	
Site of primary			
Colon	2992 (75)	453 (82.1)	<.001
Rectum	998 (25)	99 (17.9)	
Sidedness			
Left	2028 (50.8)	247 (44.75)	.02
Right	1471 (36.9)	220 (39.86)	
Other	491 (12.3)	85 (15.40)	
Charlson Comorbidity Index score			
0	2320 (58.2)	267 (48.4)	<.001
1	998 (25)	144 (26.1)	
≥2	672(16.8)	141 (25.5)	
Chemotherapy lines^c^			
1	3060 (76.7)	413 (74.8)	.92
≥2	930 (23.3)	139 (25.2)	
Year of diagnosis			
2004-2007	1836 (46)	235 (42.6)	.13
After 2007	2154 (54)	317 (57.4)	
Biologic agent use^d^			
Any	2539 (63.6)	355 (64.3)	.33
Anti-VEGF only^e^	1766 (69.6)	253 (71.3)	
Anti-EGFR only^e^	58 (2.3)	11 (3.1)	
Both^e^	715 (28.2)	91 (25.6)	

Abbreviations: EGFR, epidermal growth factor receptor; NA, not applicable; VEGF, vascular endothelial growth factor.

^a^ This table includes important clinical characteristics in the SEER Medicare database among 4542 patients (12.2% Black patients) that may be associated with survival experiences.

^b^ Served as a proxy for socioeconomic status.

^c^ At any point during the study period, not strictly a baseline covariate and treated as a time-dependent covariate in subsequent analysis.

^d^ Within 3 months of the first dose of chemotherapy, not strictly a baseline covariate and treated as a time-dependent covariate in subsequent analysis.

^e^ Percent of patients among those who received biologic agent.

### Clinical Outcome and Survival Analysis

The median OS was 17.9 (95% CI, 17.3-18.7) in patients who received biochemotherapy and 8.3 (95% CI, 9.1-9.9) months in patients who received chemotherapy (*P* < .001) (Figure and Table 2). In an unadjusted analysis, younger age (dichotomized at median) (age ≥72 years vs age <72 years, HR, 1.39; 95% CI,1.30-1.49; *P* < .001) left sidedness of tumor (right sidedness, HR, 1.27; 95% CI, 1.18-1.36; *P* < .001), well/moderate differentiation (poorly differentiated, HR, 1.45; 95% CI, 1.34-1.57; *P* < .001), 2 or more lines of chemotherapy (1 line, HR, 1.76; 95% CI, 1.62-1.90; *P* < .001), having a lower comorbidity score (score of 1, HR, 1.27; 95% CI, 1.18-1.36; *P* < .001), and being diagnosed more recently (after 2007, HR, 0.87; 95% CI, 0.82-0.94; *P* = .001), were all associated with better OS (Table 3).

**Figure.**
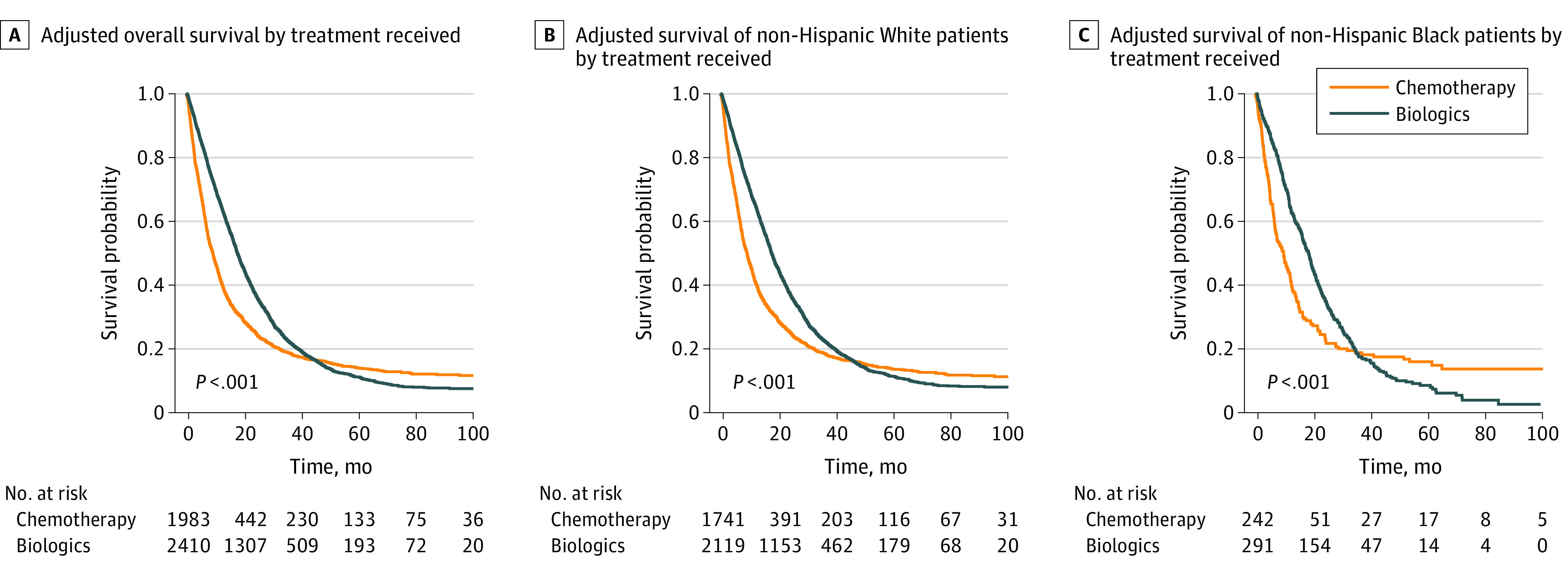
Kaplan-Meier Survival Curves for Patients Receiving Chemotherapy vs Biochemotherapy Survival curves accommodating biologic agents as a time-dependent covariate. For those individuals who did not receive biologic agents concurrently with their first chemotherapy dose, the initial follow-up time until they received biologic agents was attributed to chemotherapy. Numbers at risk were rounded to avoid fractions resulting from adjustment, and the numbers do not necessarily correspond to the actual number of patients at a given point.

**Table 2.** Survival Using Inverse Probability of Treatment Weighting

Population by racial category	Median survival in mo (95% CI)		*P* value
	Chemotherapy	Biochemotherapy	
Overall	9.1 (8.3-9.9)	17.9 (17.3-18.7)	<.001
White	9.0 (8.2-9.8)	17.8 (17.2-18.6)	<.001
Black	9.9 (7.1-12.2)	18.6 (16.4-20.3)	<.001

**Table 3.** Unadjusted Analysis of Variables With Outcome of Overall Survival

Covariate	Hazard ratio (95% CI)^a^	*P* value
Treatment group		
Chemotherapy	1 [Reference]	
Biochemotherapy	0.60 (0.55-0.64)	<.001
Race		
White	1 [Reference]	
Black	1.01 (0.91-1.12)	.83
Age, y		
<72	1 [Reference]	
≥72	1.39 (1.30-1.49)	<.001
Sex		
Male	1 [Reference]	
Female	1.08 (1. 01-1.16)	.03
Census tract median income^b^		
First quartile	1 [Reference]	
Second quartile	1.02 (0.93-1.13)	.62
Third quartile	1.00 (0.91-1.10)	.95
Top quartile	0.98 (0.89-1.08)	.71
Marital status^b^		
Single	1 [Reference]	
Married	0.97 (0.87-1.09)	.66
Separated/divorced/widowed	1.08 (0.95-1.22)	.25
Grade of differentiation^a^		
Well/moderate	1 [Reference]	
Poor	1.45 (1.34-1.57)	<.001
Location		
Colon	1 [Reference]	
Rectum	0.97 (0.90-1.05)	.51
Sidedness		
Left	1 [Reference]	
Right	1.27 (1.18-1.36)	<.001
Other	1.39 (1.25-1.55)	
Charlson Comorbidity Index score		
0	1 [Reference]	
1	1.27 (1.18-1.36)	<.001
≥2	1.39 (1.25-1.55)	
Chemotherapy lines		
≥2	1 [Reference]	
1	1.76 (1.62-1.90)	<.001
Year of diagnosis		
2007 and Before	1 [Reference]	
After 2007	0.87 (0.82-0.94)	.001

^a^ Average hazard ratio.

^b^ Multiple imputation employed.

In a model providing an overall average effectiveness of biochemotherapy, the added benefit of biologic agents was consistent with a 41% improvement in survival compared with chemotherapy alone (HR, 0.59; 95% CI, 0.55-0.64; *P* < .001). Biochemotherapy was associated with improved OS in White patients (17.8-month vs 9 months; HR, 0.59; 95% CI, 0.54-0.64; *P* < .001) and in Black patients (18.6 months vs 9.9 months; HR, 0.58; 95% CI, 0.47-0.71; *P* < .001) compared with chemotherapy (Table 3 and Table 4). All 3 comparisons were statistically significant after adjustment for multiple comparisons. There was no interaction between biochemotherapy and race.

**Table 4.** IPTW-Based Analysis of Overall Effectiveness of Biologic Agents^a^

Population by racial category	Hazard ratio^b^ (95% CI)	*P* value	Adjusted *P* value^c^
Overall	0.59 (0.55-0.64)	<.001	<.001
White	0.59 (0.54-0.64)	<.001	<.001
Black	0.58 (0.47-0.71)	<.001	<.001

Abbreviation: IPTW, inverse probability of treatment weighting (chemotherapy + biologic agents vs chemotherapy alone).

^a^ IPTW-based analysis showing overall effectiveness of biologic agents (results showing the time-varying effect of biologic agents are provided in eTable 4 in the Supplement).

^b^ Average hazard ratio.

^c^ *P* value adjusted for multiple testing using the Benjamini and Hochberg approach. For interaction between biologic agents and race, *P* = .89 (adjusted *P* > .99).

### Change in the Effectiveness of Biochemotherapy Over Time

An attenuation of the effectiveness (peak benefit at 1.3 years, eFigure in the Supplement), with crossing of the survival curves at 4 years was noted (Figure). This finding was consistent when analysis was restricted by Black or White race. To visualize the time-varying pattern of the effectiveness of biologic agents, a cumulative regression coefficient of biochemotherapy derived from a dynamic modeling approach was plotted and revealed that the effectiveness of biochemotherapy was not constant. Effectiveness noted early progressively attenuated then possibly transitioned to appear harmful as follow-up increased.

This information was integrated in our exploratory analysis using a time-varying coefficients model, ie, piecewise weighted Cox regression model using intervals determined by graphical exploration (0-12, 12-24, and >24 months). These intervals happen to be clinically interpretable because 1-year and 2-year survival probabilities are considered when making clinical decisions. The same finding was apparent overall as well as within each racial and ethnic group (eTable 4 in the Supplement).

### Sensitivity Analysis

Because the main analysis excluded patients whose treatment with biologic agents was initiated after 3 months of start of chemotherapy, a sensitivity analysis was performed censoring those patients at the time of initiating treatment with biologic agents and using inverse probability of censoring weighting to account for the possibility of selection bias due to this artificial censoring. The sensitivity analysis revealed that the main finding of survival benefit across racial groups did not change; even the effectiveness of biologic agents became more pronounced (eTables 5 and 6 in the Supplement).

## Discussion

Phase 3 RCTs form the reference standard of evidence generation and regulatory drug approval. However, Phase 3 RCTs have been criticized for the lack of population representation of the real world and for the highly selected patient population that is enrolled.^13^ One approach to address this concern is the concept of comparative effectiveness research. The core question of comparative effectiveness research is which treatment works best, for whom, and under what circumstances.^35^ An additional major limitation of RCTs in the US has been the lack of representation of racial and ethnic minority populations, which as of 2019 have been limited to 10%^13,14^ of patient accruals, whereas per the 2020 census, racial and ethnic minority populations constitute 39% of the US population.^36^ Among patients with advanced noncurable cancers, White patients experience a more favorable outcome compared with Black and Hispanic patients.^37,38,39^ The probable reasons vary, ranging from better access to care for White people in the US (remediable by adequate policy and administrative interventions) to the ongoing debate about biologic factors and race and ethnicity in disease outcomes.

To date, there is no large population-based study informing the treating clinician whether a particular intervention will be equally beneficial to patients who identify as being a member of a racial minority group as to White patients with mCRC. We report for the first time to date that Black patients experience a survival advantage (with a 42% improvement in survival) from the addition of biologic agents to cytotoxic chemotherapy similar to the survival benefit found for White patients. Two notable findings have been borne out by this study. The first is that the median OS with biochemotherapy among Black patients is similar to the median OS with biochemotherapy among White patients (OS for Black patients was 18.6 months and OS for White patients was 17.8 months). The second finding is that the rates of receipt of biologic agents are similar among both racial groups. This finding may suggest that given the appropriate medical intervention unencumbered by access to care or medication coverage, Black patients can experience the same clinical benefit as White patients. Thus, the driving force behind the lower OS among Black patients may be the lack of adequate access to high-quality health care. Data from SEER that focuses on outcomes-enriched data clearly highlights the lower OS rates among Black patients.^39^

To replicate real world practice, we defined biochemotherapy as receipt of biologic agents within 3 months of the start of chemotherapy. The start of therapy with biologic agents is often delayed in routine clinical practice because of a lack of availability of genomic profile data, missing required laboratory tests, delays in obtaining preauthorization, among other reasons. Approximately 23% of the patients received therapy with biologic agents after 3 months, and those patients were not included in the main analysis owing to concerns that inclusion may unfairly bias the results against the potential benefit of biologic agent. However, results of a sensitivity analysis by censoring such patients at the time of late initiation of biologic agents did not affect our main conclusion.

The conclusion from a prior study^20^ that reported a differential benefit of biologic agents in Black patients compared with White patients was not replicated in this study. One likely explanation may be that the prior report was from a large single-center patient experience, and compliance and insurance information were not captured. In contrast, the current SEER-Medicare linked data set included patients exclusively with Medicare coverage, potentially ensuring timely and appropriate access to all therapy, intervention, and drugs. This study also encompassed a broader patient experience from across the US spanning multiple states, and so experiences may differ. In addition, unlike the current Medicare eligible population (median age 73 years), the prior report population was younger with a median age of 63 years.

### Limitations

Our data has limitations deserving discussion. The SEER-Medicare linked database lacks information on the status of tumor mutations, including *KRAS* and *NRAS* (biomarkers of exclusion from anti-EGFR therapy), and *BRAF* (biomarker of poor prognosis). A previous study^40^ reported that the incidence of *KRAS* sequence variation was 61% among Black patients and 49% among White patients; and that *BRAF* V600E mutation was 2.8% among Black patients and 8.5% among White patients. However, while incorporation of these genomic biomarkers may allow for a more comprehensive analysis; it may be unlikely that the missing information would affect our findings. Second, the database has limitations, some of which we have tried to address, and which have been discussed in previous publications.^41,42^ For instance, the SEER Medicare population is better insured, more affluent, more urban than the rest of the country. However, this does not limit the importance and relevance of our research. In addition, most data on race are based on patient self-report, and it may be argued that the definition of race is undergoing a transformation, based on genomic and ancestry information.^43,44,45^

## Conclusions

In the comparative effectiveness analysis of data from the SEER-Medicare database, the addition of biologic drugs to chemotherapy was found to be associated with a meaningful improvement in OS among Medicare recipients, was observed early, and was uniform across Black and White patients; however, caution is warranted. Such large database analysis is not a substitute for the low representation of racial and ethnic minority groups in RCTs. Until racial and ethnic minority groups are equitably included in RCTs, oncologists may prescribe biologic agents for patients with mCRC with reasonable faith in their early benefit, regardless of race.

## Notes

Supplement. **eFigure.** Cumulative Regression Coefficients and 95% Pointwise Confidence Interval Showing the Time-Varying Effect of Biologics for the Entire Population **eTable 1.** Frequency of Patients Receiving Biologics Based on the Timing of Start of Biologic Therapy Relative to Chemotherapy **eTable 2.** Distribution of Chemotherapy Agents (N = 5617) **eTable 3.** Distribution of Biologics Agents (N=3969) **eTable 4.** Time-Varying Effect: IPTW Based Analysis Overall and Across the Races **eTable 5.** Overall Effect: Inverse Probability of Treatment and Censoring Weighted Sensitivity Analysis **eTable 6.** Time-Varying Effect: Inverse Probability of Treatment and Censoring Weighted Sensitivity Analysis
