# Supplementary material for: Comparative Effectiveness of Biologic Agents Among Black and White Medicare Patients in the US With Metastatic Colorectal Cancer
Source: JAMA Netw Open. 2021 Dec 15;4(12):e2136378. doi: 10.1001/jamanetworkopen.2021.36378 (PMC8674750; doi:10.1001/jamanetworkopen.2021.36378)
Supplement: Supplement. — eFigure. Cumulative Regression Coefficients and 95% Pointwise Confidence Interval Showing the Time-Varying Effect of Biologics for the Entire Population eTable 1. Frequency of Patients Receiving Biologics Based on the Timing of Start of Biologic Therapy Relative to Chemotherapy eTable 2. Distribution of Chemotherapy Agents (N = 5617) eTable 3. Distribution of Biologics Agents (N=3969) eTable 4. Time-Varying Effect: IPTW Based Analysis Overall and Across the Races eTable 5. Overall Effect: Inverse Probability of Treatment and Censoring Weighted Sensitivity Analysis eTable 6. Time-Varying Effect: Inverse Probability of Treatment and Censoring Weighted Sensitivity Analysis [file jamanetwopen-e2136378-s001.pdf]

Supplemental Online Content

Goel S, Negassa A, Acuna-Villaorduna A. Comparative effectiveness of biologic agents among Black and White Medicare patients in the US with metastatic colorectal cancer. *JAMA Netw Open.* 2021;4(12):e2136378. doi:10.1001/jamanetworkopen.2021.36378

eFigure. Cumulative Regression Coefficients And 95% Pointwise Confidence Interval Showing The Time-Varying Effect Of Biologics For The Entire Population
eTable 1. Frequency of Patients Receiving Biologics Based on the Timing of Start of Biologic Therapy Relative to Chemotherapy
eTable 2. Distribution of Chemotherapy Agents (N = 5617)
eTable 3. Distribution of Biologics Agents (N=3969)
eTable 4. Time-Varying Effect: IPTW Based Analysis Overall and Across the Races
eTable 5. Overall Effect: Inverse Probability of Treatment and Censoring Weighted Sensitivity Analysis
eTable 6. Time-Varying Effect: Inverse Probability of Treatment and Censoring Weighted Sensitivity Analysis

This supplemental material has been provided by the authors to give readers additional information about their work.

© 2021 Goel S et al. *JAMA Network Open.*

e Figure 1

a. Cumulative regression coefficients and 95% pointwise confidence interval showing the time-varying effect of biologics for the entire population.

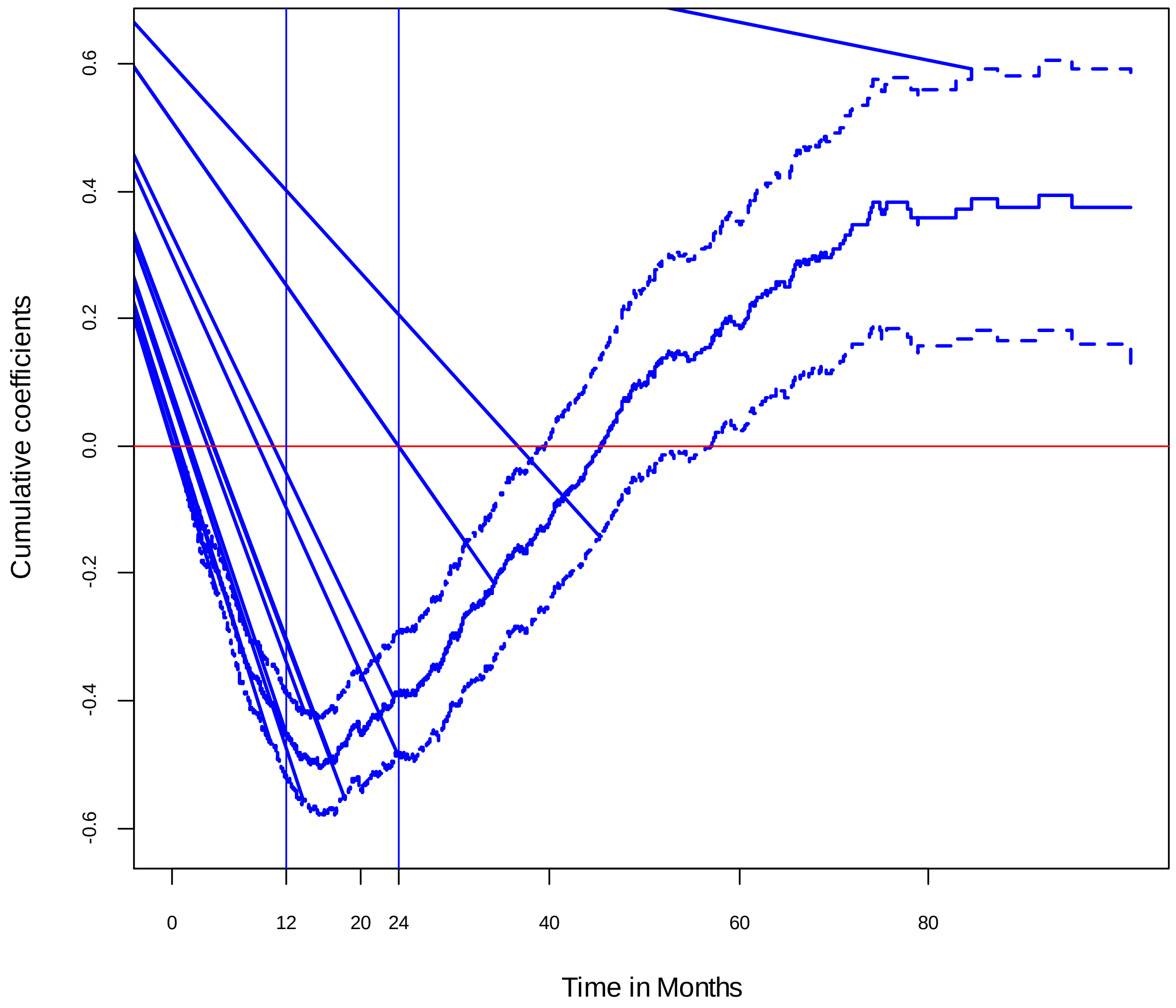

© 2021 Goel S et al. *JAMA Network Open.*

b. Cumulative regression coefficients and 95% pointwise confidence interval showing the time-varying effect of biologics among Non-Hispanic White Patients.

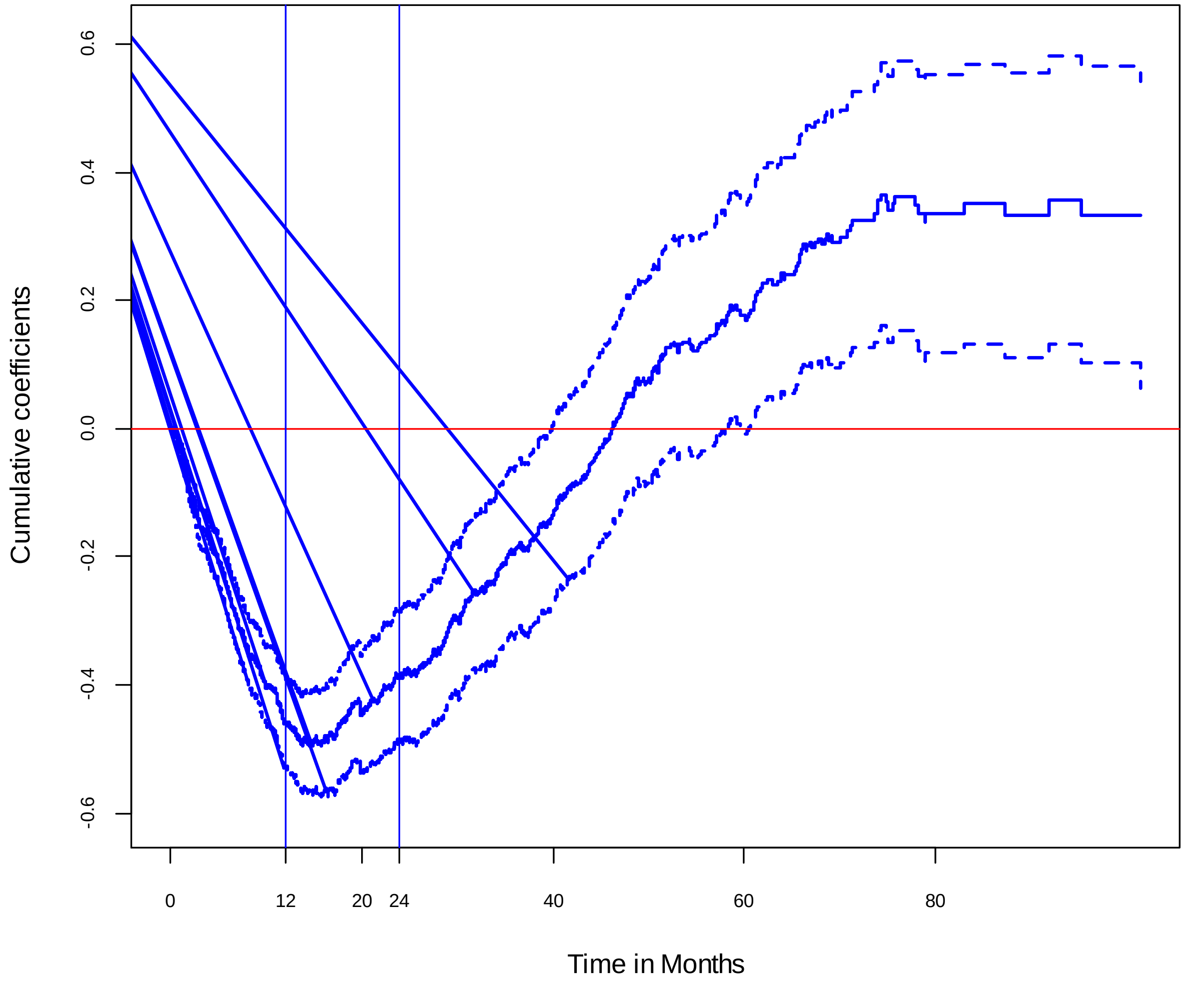

© 2021 Goel S et al. *JAMA Network Open.*

c. Cumulative regression coefficients and 95% pointwise confidence interval showing the time-varying effect of biologics among Non-Hispanic Black Patients.

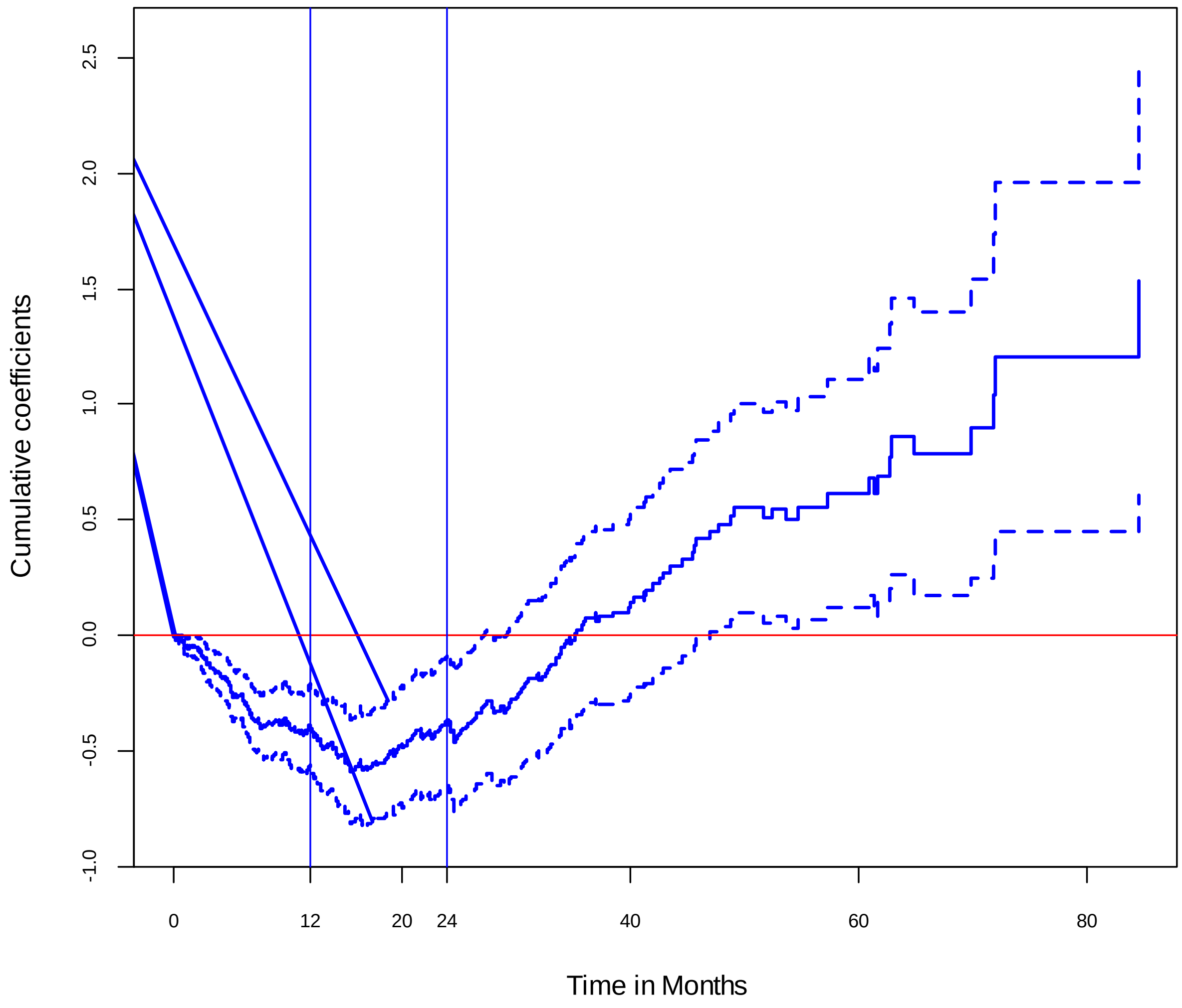

Cumulative regression coefficients and 95% pointwise confidence interval showing the time-varying effect of biologics for the entire population (1a), non-Hispanic Whites (1b), and non-Hispanic Blacks (1c). Each display is split into three sections along the x axis, corresponding to the suggested time intervals: 0–12 , >12–24 and >24 months.

© 2021 Goel S et al. *JAMA Network Open.*

**eTable 1: Frequency of patients receiving biologics based on the timing of start of biologic therapy relative to chemotherapy.**			
	NHW	NHB	Overall
Biologics before chemotherapy	4.2	5.5	4.3
Biologics with chemotherapy	43.4	49.8	44.2
Biologics within 1 month of chemotherapy	18.7	13.2	18.0
Biologics within 1-2 months of chemotherapy	6.6	7.7	6.8
Biologics within 2-3 months of chemotherapy	3.9	3.8	3.9
Biologics after > 3 months of chemotherapy	23.2	20.0	22.8
Total	100.0	100.0	100.0

NHW: Non-Hispanic White, NHB: Non-Hispanic Black

Table displays percent of patients among NHW and NHB, and overall, who received biochemotherapy based on the timing of start of biologic therapy relative to start of chemotherapy

© 2021 Goel S et al. *JAMA Network Open.*

**eTable 2: Distribution of chemotherapy agents (N = 5617)**			
Chemotherapy	Non-Hispanic White 4944 (%)	Non-Hispanic Black 673 (%)	Total
J9263: Oxaliplatin	3,569 (72.2)	469 (69.7)	4,038 (71.9)
J8520: Capecitabine, 150 mg	24 (0.5)	3 (0.4)	27 (0.5)
J8521: Capecitabine, 500 mg	12 (0.2)	1 (0.2)	13 (0.2)
J9206: Irinotecan	2,443 (49.4)	339 (50.4)	2,782 (49.5)
J9190: Fluorouracil	3,995 (80.8)	535 (79.5)	4,530 (80.7)

Table displays details of receipt of chemotherapy drugs by race and overall

© 2021 Goel S et al. *JAMA Network Open.*

**e Table 3: Distribution of biologics agents (N=3969)**			
Biologics	Non-Hispanic White n= 3493 (%)	Non-Hispanic Black n= 476 (%)	Total
J9035: Bevacizumab	3,024 (86.57)	405 (85.08)	3,429 (86.39)
J9055: Cetuximab	1,120 (32.06)	143 (30.04)	1,263 (31.82)
J9303: Panitumumab	222 (6.36)	27 (5.67)	249 (6.27)
J0178: Aflibercept	0 (0)	0 (0)	0 (0)
J9308: Ramucirumab	0 (0)	0 (0)	0 (0)

Table displays details of receipt of biologic agents, by race and overall

© 2021 Goel S et al. *JAMA Network Open.*

eTable 4. Time-varying effect: IPTW based analysis overall and across the races					
Group	Period (months)	Hazard ratio [a]	95% CI	p-value	Adjusted [b] p-value
Overall	0 – 12	0.45	0.41 – 0.49	<0.001	<0.001
	>12 – 24	0.96	0.81 – 1.13	0.63	1
	>24	1.88	1.48 – 2.38	<0.001	<0.001
Non-Hispanic White	0 – 12	0.45	0.41 – 0.49	<0.001	<0.001
	>12 – 24	1	0.83 – 1.20	0.99	1
	>24	1.87	1.45 – 2.42	<0.001	<0.001
Non-Hispanic Black	0 – 12	0.46	0.36 – 0.60	<0.001	<0.001
	>12 – 24	0.73	0.47 – 1.12	0.15	0.60
	>24	1.93	1.12 – 3.32	0.02	0.09

[a] Average Hazard Ratio based on time-varying coefficients model. IPTW = Inverse probability of treatment weighting (biochemotherapy vs. chemotherapy). [b]*p*-value adjusted for multiple testing using the Benjamini and Hochberg approach.

IPTW based analysis overall and across the races, shown across three different time points. There is a clear overall improvement in OS by the use of biologics; however, it is time-varying, with a detrimental effect at > 24 months.

© 2021 Goel S et al. *JAMA Network Open.*

**eTable 5. Overall effect: Inverse probability of treatment and censoring weighted sensitivity analysis**				
Group	Hazard ratio[a]	95% CI	p-value	Adjusted[b] p-value
Overall	0.49	0.45 – 0.53	<0.001	<0.001
Non-Hispanic White	0.49	0.45 – 0.53	<0.001	<0.001
Non-Hispanic Black	0.51	0.41 – 0.63	<0.001	<0.001

[a] Average Hazard Ratio. **[b]***p*-value adjusted for multiple testing using the Benjamini and Hochberg approach (biochemotherapy vs chemotherapy). Adjusted *p*-value for interaction between biologics and race= 0.61.

© 2021 Goel S et al. *JAMA Network Open.*

**e Table 6. Time-varying effect: Inverse probability of treatment and censoring weighted sensitivity analysis.**					
Group	Period (months)	Hazard ratio [a]	95% CI	p-value	Adjusted [b] p-value
Overall	0 – 12	0.36	0.33 – 0.39	<0.001	<0.001
	>12 – 24	0.92	0.79 – 1.06	0.26	0.61
	>24	1.74	1.45 – 2.08	<0.001	<0.001
Non-Hispanic White	0 – 12	0.35	0.32 – 0.39	<0.001	<0.001
	>12 – 24	0.95	0.81 – 1.11	0.53	0.61
	>24	1.66	1.37 – 2.00	<0.001	<0.001
Non-Hispanic Black	0 – 12	0.38	0.29 – 0.49	<0.001	<0.001
	>12 – 24	0.74	0.49 – 1.10	0.14	0.55
	>24	2.57	1.41 – 4.67	0.002	0.01

[a] Average Hazard Ratio based on time-varying coefficients model. Patients who initiated biologics after 3 months of the start of chemotherapy were artificially censored at the time of initiating biologics. [b] *p*-value adjusted for multiple testing using the Benjamini and Hochberg approach.

© 2021 Goel S et al. *JAMA Network Open.*
